# Advancing Zinc–Manganese Oxide Batteries: Mechanistic Insights, Anode Engineering, and Cathode Regulation

**DOI:** 10.3390/nano15181439

**Published:** 2025-09-18

**Authors:** Chuang Zhao, Yiheng Zhou, Yudong Liu, Bo Li, Zhaoqiang Li, Yu Zhang, Deqiang Wang, Ruilin Qiu, Qilin Shuai, Yuan Xue, Haoqi Wang, Xiaojuan Shen, Wu Wen, Di Wu, Qingsong Hua

**Affiliations:** 1 Department of Physics, Faculty of Arts and Sciences, Beijing Normal University, Zhuhai 519087, China; 202321220017@mail.bnu.edu.cn (C.Z.); 18925636636@163.com (Y.Z.); 13510028032@163.com (Y.L.); ciaczyu@mail.ustc.edu.cn (Y.Z.); dqwang@mail.bnu.edu.cn (D.W.); 202331220017@mail.bnu.edu.cn (R.Q.); 202131220021@mail.bnu.edu.cn (Q.S.); 202421101102@mail.bnu.edu.cn (Y.X.); wanghaoqi@bjast.ac.cn (H.W.); 2 Success Bio-Tech Co., Ltd., Jinan 250101, China; tbyilu@163.com; 3 School of Materials Science & Engineering, Jiangsu University, Zhenjiang 212013, China; xiaojuanshen@ujs.edu.cn; 4 Instrumentation and Service Center for Science and Technology, Beijing Normal University, Zhuhai 519087, China; 5 Physics Group, Sun Yat-sen Memorial Secondary School, Zhongshan 528454, China

**Keywords:** Zn-ion battery, manganese oxide cathode, zinc anode, zinc-manganese oxide battery, reaction mechanism

## Abstract

Rechargeable aqueous Zn-MnO_2_ batteries are positioned as a highly promising candidate for next-generation energy storage, owing to their compelling combination of economic viability, inherent safety, exceptional capacity (with a theoretical value of ≈308 mAh·g^−1^), and eco-sustainability. However, this system still faces multiple critical challenges that hinder its practical application, primarily including the ambiguous energy storage reaction mechanism (e.g., unresolved debates on core issues such as ion transport pathways and phase transition kinetics), dendrite growth and side reactions (e.g., the hydrogen evolution reaction and corrosion reaction) on the metallic Zn anode, inadequate intrinsic electrical conductivity of MnO_2_ cathodes (≈10^−5^ S·cm^−1^), active material dissolution, and structural collapse. This review begins by systematically summarizing the prevailing theoretical models that describe the energy storage reactions in Zn-Mn batteries, categorizing them into the Zn^2+^ insertion/extraction model, the conversion reaction involving MnO_x_ dissolution–deposition, and the hybrid mechanism of H^+^/Zn^2+^ co-intercalation. Subsequently, we present a comprehensive discussion on Zn anode protection strategies, such as surface protective layer construction, 3D structure design, and electrolyte additive regulation. Furthermore, we focus on analyzing the performance optimization strategies for MnO_2_ cathodes, covering key pathways including metal ion doping (e.g., introduction of heteroions such as Al^3^^+^ and Ni^2^^+^), defect engineering (oxygen vacancy/cation vacancy regulation), structural topology optimization (layered/tunnel-type structure design), and composite modification with high-conductivity substrates (e.g., carbon nanotubes and graphene). Therefore, this review aims to establish a theoretical foundation and offer practical guidance for advancing both fundamental research and practical engineering of Zn-manganese oxide secondary batteries.

## 1. Introduction

Against the backdrop of global sustainable development, energy and environmental issues have emerged as one of the core challenges confronting human society [1,2]. These pressing issues have spurred significant scientific efforts towards the exploration and development of novel clean energy sources, positioning it as a critical research frontier [3,4,5,6]. However, the inherent volatility and intermittency of renewable energy sources (wind, solar, and geothermal energy) have rendered the development of high-efficiency energy storage devices a critical scientific issue demanding urgent resolution. Lithium-ion batteries (LIBs) are widely regarded as the foundation of the modern electrochemical energy storage landscape due to their unparalleled commercial success, which is evident in their application across dominant sectors including electric vehicles and portable electronics [7,8,9]. Nevertheless, the constraints of their relatively low theoretical specific capacity (≈372 mAh·g^−1^) and the cost escalation issues arising from the scarcity of lithium resources (with a crustal abundance of merely 0.0065%) have motivated the search for alternative energy storage systems. These new systems aim to offer advantages in both cost-effectiveness and higher theoretical capacity, and their development has become a prominent focus in energy science [10,11,12,13,14].

In recent years, a variety of representative energy storage systems have been developed, including sodium-ion batteries (SIBs), zinc-ion batteries (ZIBs), and zinc–manganese oxide batteries, among others. These systems, each with distinct advantages and limitations, have significantly broadened the technological landscape of energy storage. Lithium-ion batteries (LIBs), characterized by high energy density (150–250 Wh/kg) and operating voltages (3.0–4.2 V), remain the dominant technology for high-performance applications such as electric vehicles (EVs) and portable electronics. However, they are confronted with challenges related to high material costs—stemming from the scarcity of critical elements like lithium and cobalt—and inherent safety hazards associated with flammable organic electrolytes. As low-cost alternatives, SIBs leverage sodium’s earth abundance, exhibiting moderate energy density (100–160 Wh/kg) and compatibility with aqueous electrolytes to mitigate safety concerns, though their commercialization is hindered by lower energy density stemming from the larger Na^+^ ionic radii. ZIBs and Zn-MnO_2_ batteries, conversely, prioritize safety and affordability: both utilize non-flammable aqueous electrolytes and low-cost Zn anodes ($2–5/kg), with Zn-MnO_2_ batteries offering unparalleled cost competitiveness (<$10/kWh) suitable for low-cost grid storage, while ZIBs advance in cycle life and flexibility for wearable electronics. Environmentally, ZIBs and Zn-MnO_2_ batteries excel via abundant Zn/Mn resources and high recyclability (>90%), whereas LIBs and SIBs face sustainability constraints from critical material reliance and underdeveloped recycling infrastructure. Research frontiers include solid-state electrolytes for LIBs to enhance safety, Mn-based cathode engineering for ZIBs to boost voltage, and hard carbon anode optimization for SIBs to improve energy density, collectively shaping their future roles in the energy transition.

Within the aforementioned energy storage systems, zinc-ion batteries (ZIBs) have attracted considerable research interest within the energy storage research community by virtue of their unique combination of advantages [15,16,17]. Compared with lithium metal (crustal abundance: 0.0065%), zinc resources are more abundant (crustal abundance: ≈0.0076%), exhibit lower raw material costs (zinc prices are approximately 1/5 of lithium prices), and demonstrate superior environmental friendliness (non-toxic and harmless) [18,19]. Additionally, the standard electrode potential of zinc metal (−0.76 V vs. SHE) enables its compatibility with aqueous electrolyte systems, significantly enhancing the safety performance of the batteries. However, Zn-MnO_2_ batteries still face multiple key scientific challenges [20,21,22,23]: First, the phase transition behavior and disproportionation reaction of MnO_2_ can induce the dissolution of manganese species and structural collapse of materials, significantly reducing the cycling stability and lifespan of electrode materials. Second, the synergistic effect of the low intrinsic electrical conductivity of manganese oxides (≈10^−5^ S·cm^−1^) and the large ionic radius of Zn^2^^+^ (0.74 Å) leads to sluggish electrochemical reaction kinetics. Third, there remains a lack of unified understanding regarding energy storage reaction mechanisms, with extensive debates persisting on critical issues such as intercalation/deintercalation mechanisms and conversion reaction pathways [24].

Regarding the above-mentioned issues, researchers have conducted a series of explorations to enhance electrochemical performances. Using advanced characterization techniques, they have identified the energy storage mechanisms as the Zn^2^^+^ intercalation–deintercalation mechanism, the proton/Zn^2^^+^ co-intercalation mechanism, and the MnO_x_ dissolution–deposition mechanism. Notably, the actual energy storage process often involves multiple mechanisms, with this dependence determined by the electrode materials and electrolyte. For anode design, with the aim of avoiding Zn dendrite formation and achieving uniform ion flux, numerous protection and modification strategies have been developed, including three-dimensional configuration engineering and protective layers based on metal oxides, metal sulfides, artificial solid electrolyte interphases (SEI), and even MOF functional layers. For cathode optimization, effective strategies to enhance electrochemical kinetics for energy storage include metal ion doping (e.g., Na^+^, K^+^), defect engineering (including oxygen defects, cation defects, and heteroatom (N, P) doping), and compositing with conductive materials (e.g., various carbon materials, conductive polymers, etc.). Although significant progress has been achieved in the above areas, systematic reviews on this system remain relatively scarce [25,26], necessitating an urgent need for systematic summarization and collation of research progress in this field.

**Figure 1:**
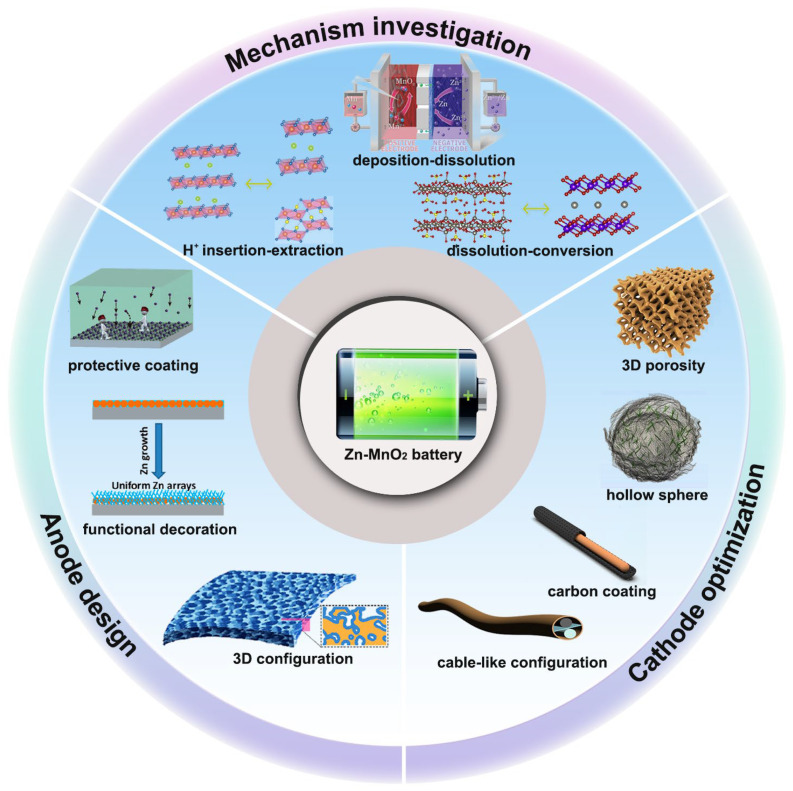
Recent advances in Zn-MnO_2_ batteries towards mechanism investigation, anode design, and cathode optimization.

As depicted in Figure 1, this review centers on the key scientific issues of Zn-Mn secondary batteries, covering three core directions: energy storage mechanism analysis, Zn anode protection strategies, and optimization design of Mn-based cathode materials. Specifically, it systematically elaborates on typical energy storage models, including the Zn^2^^+^ intercalation–deintercalation mechanism, proton-assisted conversion reaction mechanism, proton/Zn^2^^+^ co-intercalation mechanism, and MnO_x_ dissolution–deposition mechanism; summarizes key strategies for Zn anode surface protective layer construction (e.g., metallic/non-metallic coatings, organic polymer films) and structural optimization (e.g., 3D current collector design, gradient concentration regulation); and emphasizes the analysis of performance optimization pathways for Mn-based cathode materials, including metal ion doping (e.g., Al^3^^+^, Ni^2^^+^), defect engineering (oxygen vacancy/cation vacancy regulation), structural optimization (layered/tunnel-type structure design), and composite modification with high-conductivity substrates (e.g., carbon nanotubes, graphene). Consequently, this review is intended to furnish a valuable framework, derived from a systematic summarization of the field, to guide the future development of Zn-Mn secondary batteries from basic scientific exploration to scale-up engineering applications.

## 2. Reaction Mechanisms

Electrode reaction mechanisms are intimately associated with the local chemical and electrochemical environments, which are governed by electrolyte composition and electrode structural evolution. Discussing charge storage mechanisms in the absence of specific electrolyte environments and charge–discharge conditions is ambiguous and misleading. Accordingly, this paper systematically summarizes and critically discusses the charge storage mechanisms of manganese dioxide under different electrolyte environments.

### 2.1. Zn^2+^ Insertion/Extraction Mechanism

**Figure 2:**
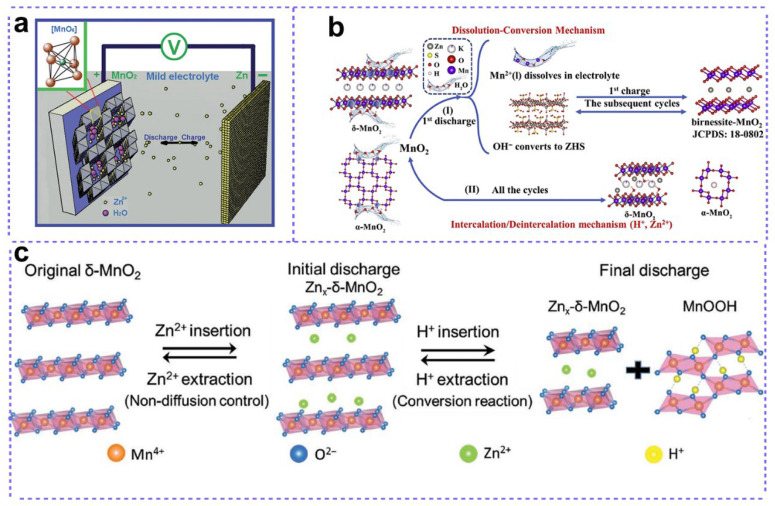
Zn-MnO_2_ batteries with different energy storage mechanisms. (**a**) Zn^2+^ insertion/extraction mechanism. Adapted from [27], with permission from *Angew. Chem.*, 2012. (**b**) Dissolution–conversion mechanism. Adapted from [35], with permission from *Materials Today Energy*, 2020. (**c**) H^+^/Zn^2+^ co-insertion/extraction mechanism. Adapted from [32], with permission from *Adv. Mater.*, 2019.

Kang reported the fabrication of a rechargeable zinc ion batteries by employing zinc metal as an anode, α-MnO_2_ as a cathode, and Zn^2+^ containing mild aqueous solution (Zn(NO_3_)_2_ or ZnSO_4_) as an electrolyte in 2012 [27]. The robust tunnel framework of α-MnO_2_ can readily accommodate the reversible intercalation and deintercalation of Zn^2+^ ions during the discharge and charge cycles, as illustrated in Figure 2a. The insertion of Zn^2+^ ions into α-MnO_2_ was confirm by XRD and XPS, in which Zn signals were detected in Zn^2+^ ion insertion states [28]. Based on these results, the reaction mechanisms were proposed as follows:

$${Zn}^{2+}+2e^{-}+2MnO_{2}↔Zn{Mn}_{2}O_{4}$$

### 2.2. H^+^ Related Conversion Mechanism

Another alternative conversion mechanism was proposed by Liu and co-workers in 2016 [29]. They constructed an aqueous Zn/MnO_2_ battery by using α-MnO_2_ nanofiber as a cathode, Zn metal as an anode and mild ZnSO_4_ solution as an electrolyte, but with some MnSO_4_ additive to alleviate the dissolution of Mn^2+^ into the electrolyte. The battery exhibited an operating voltage of 1.44 V, with a high capacity retention of 92.2% after 5000 cycles at 5 C. Further TEM, XRD, and STEM-EDS investigations of the discharged products indicate that monoclinic MnOOH phase was formed. The MnO_2_ likely reacted with a proton from water to form MnOOH, and the OH^−^ reacted with ZnSO_4_ and H_2_O in the electrolyte to form ZnSO_4_[Zn(OH)_2_]_3_·xH_2_O. Instead of Zn^2+^ insertion into MnO_2_, the conversion mechanism between MnOOH and MnO_2_ in this system was proposed as follows [30]:

$$\begin{array}{c} H_{2}O↔H^{+}+{OH}^{-}\\ H^{+}+e^{-}+MnO_{2}↔MnOOH\\ {\frac{1}{2}Zn}^{2+}+{OH}^{-}+\frac{1}{6}ZnSO_{4}+{\frac{x}{6}H}_{2}O↔\frac{1}{6}ZnSO_{4}[Zn({OH)}_{2}{]}_{3}\cdot xH_{2}O \end{array}$$

### 2.3. Zn^2+^/H^+^ Co-Insertion Mechanism

In the recent investigations, researchers found that both the H^+^ related conversion reaction and Zn^2+^ insertion reaction occurred in some zinc manganese battery systems [31]. For example, Liu’s group [32] reported a joint charge storage mechanism in a layered δ-MnO_2_ cathode with a Zn(TFSI)_2_-based electrolyte (Figure 2c). During discharge processes, the fast Zn^2+^ intercalation in δ-MnO_2_ with a nondiffusion-controlled process occurred in the first discharge step before 1.43 V, then the diffusion-controlled H^+^ conversion reaction dominated the deeper discharge process. Owing to the joint charge storage processes, the Zn-δ-MnO_2_ battery exhibited a high specific capacity of 137 mAh g^−1^ at an ultrahigh rate of 20 C, accompanied with a high capacity retention of 93% after 4000 cycles.

$$\begin{array}{c} MnO_{2}+x{Zn}^{2+}+2xe^{-}↔Zn_{x}MnO_{2}\;(Nondiffusion-controlled\;reaction)\\ H_{2}O↔H^{+}+{OH}^{-}\\ H^{+}+e^{-}+MnO_{2}↔MnOOH\left(diffusion-controlled\;reaction\right)\\ {\frac{1}{2}Zn}^{2+}+{OH}^{-}+\frac{1}{6}Zn({TFSI)}_{2}+{\frac{x}{6}H}_{2}O↔\frac{1}{6}Zn({TFSI)}_{2}[Zn({OH)}_{2}{]}_{3}\cdot xH_{2}O \end{array}$$

More recently, Ji’s group also proposed this H^+^/Zn^2+^ co-insertion mechanism in an α-MnO_2_ cathode [33]. However, the Zn^2+^ insertion was revealed to be less reversible than H^+^ insertion due to the formation of an irreversible ZnMn_2_O_4_ layer outside the α-MnO_2_. The H^+^ insertion dominated the charge storage process during the repeated charge/discharge processes.

### 2.4. MnO_2_ Deposition/Dissolution Mechanism

A common feature across these three mechanisms is the Mn^3+^ and Mn^4+^ redox couple, which involves a single-electron transfer reaction and affords a theoretical specific capacity of 308 mAh g^−1^. Recently, a new charge storage mechanism was reported by more and more groups (Figure 2b) [34,35], which focused on a two-electron redox reaction between MnO_2_ and Mn^2+^, corresponding to a much higher theoretical capacity of 616 mAh g^−1^.

$${4H}^{+}+{2e}^{-}+MnO_{2}↔Mn^{2+}+2H_{2}O$$

This mechanism often occurred in a mildly acidic electrolyte, with the repeated deposition and dissolution of MnO_2_ on the cathode into the electrolyte. For example, Balland’s group reported the fabrication of a Zn/MnO_2_ battery consisting of an electrodeposited MnO_2_ cathode and a weak Brønsted acid-based electrolyte [36]. This battery displayed a record gravimetric capacity of 450 mAh g^−1^ at 1.6 A g^−1^, with a MnO_2_ utilization of 84% and a coulombic efficiency of nearly 100%.

## 3. Zinc Anode Design

Metallic zinc offers key attributes, including low cost, environmental friendliness, and low flammability, making it an excellent anode for zinc–manganese battery systems. However, its practical application is still hindered by several knotty problems: (1) the formation of zinc dendrites during repeated plating/stripping processes, which could result in the short circuit of the battery by piercing the separator; (2) the side reactions (including chemical corrosion, hydrogen evolution, etc.), which could lead to poor coulombic efficiency and severe capacity loss. Considering this case, the recent advances in promoting the properties of zinc anodes are summarized in this section.

### 3.1. Functional Coating on Zn Anode

Constructing protective coatings and buffer layers on the surface of zinc anodes is proven to be an effective strategy for stabilizing zinc metal. By engineering functional protective layers, the interfacial dynamics between the electrolyte and the Zn anode can be regulated, promoting homogeneous Zn deposition and stripping behavior, which is critical for achieving high coulombic efficiency and extended cycling stability [37].

**Figure 3:**
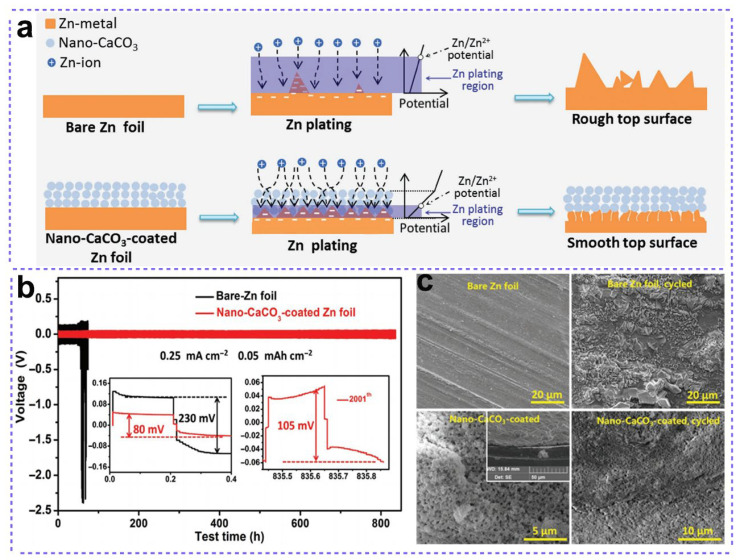
A Zn anode featuring a nano-CaCO_3_ protective layer to enhance the longevity of Zn-MnO_2_ batteries. (**a**) Schematic illustrations depicting the morphological evolution of bare and nano-CaCO_3_-coated zinc foils during repeated zinc stripping/plating cycles. (**b**) Characteristic galvanostatic charge–discharge (GCD) curves of Zn|ZnSO_4_ + MnSO_4_|Zn symmetric cells utilizing bare and nano-CaCO_3_-coated Zn electrodes. (**c**) Scanning electron microscopy (SEM) images of bare and nano-CaCO_3_-coated Zn foils before and after 100 stripping or plating cycles. Adapted from [38], with permission from *Adv. Energy Mater.*, 2018.

**Figure 4:**
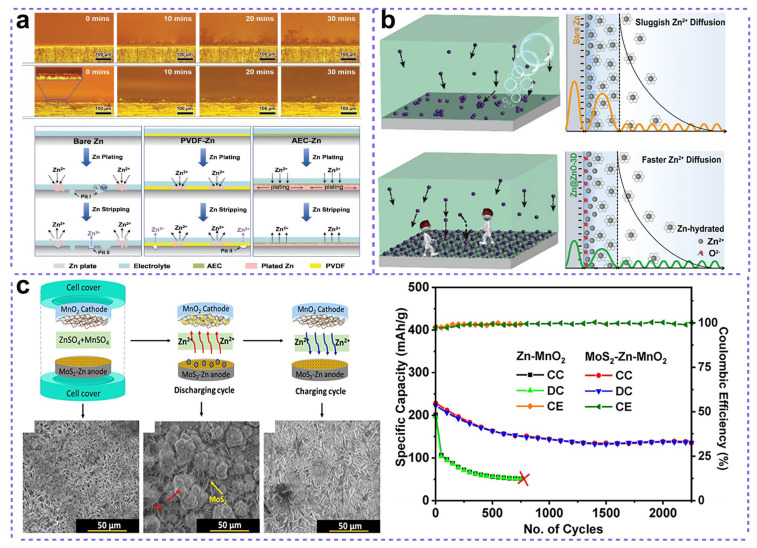
(**a**) The zinc electrodeposition behaviors of bare Zn plate and TiO_2_@PVDF anti-corrosion layer-protected Zn plate. Adapted from [39], with permission from *Adv. Funct. Mater.*, 2020. (**b**) The nanoporous ZnO architecture coating on Zn as protective layer. Adapted from [41], with permission from *Energy Environ. Sci.*, 2020. (**c**) Schematic for the charging and discharging mechanism and electrochemical performance of a MoS_2_ modified Zn//MnO_2_ battery. Adapted from [43], with permission from *ACS Appl. Mater.*, 2020.

In 2018, Liang et al. constructed nanoporous CaCO_3_ coating on the surface of a zinc anode to enable long-life aqueous rechargeable zinc manganese batteries (Figure 3), which acted as a buffer layer to guide the uniform plating of Zn metals during cycling [38]. As shown in Figure 3a, owing to its high porosity, the nano-CaCO_3_ coating facilitates the efficient permeation of the aqueous electrolyte, thereby ensuring a comparatively uniform electrolyte flux and Zn plating rate across the entire Zn foil surface. This nanoporous CaCO_3_ coating on Zn operated easily, and involved the preparation of a slurry containing commercialized CaCO_3_ particles and PVDF, followed by the casting and drying steps. During electrodeposition, Zn nucleation was guided into the nanopores of the CaCO_3_ coating. This insulating property of the nanocoating induced a significant potential gradient across it. Consequently, Zn^2+^ reduction was energetically favorable only at the interface near the Zn foil, leading to a position-selective plating process confined to this region. As a result, the nano-CaCO_3_-coated Zn cell exhibited significantly lower polarization in comparison to the bare Zn counterpart (80 vs. 230 mV, Figure 3b), even at the initial stage. A “CaCO_3_/Zn flake/Zn foil” sandwich structure was finally formed, instead of Zn protrusions/dendrites. The assembled Zn/ZnSO_4_ + MnSO_4_/CNT/MnO_2_ battery can deliver a reversible capacity of 177 mAh g^−1^ after 1000 cycles at 1 A g^−1^, which was much higher than the battery with a bare Zn anode (124 mAh g^−1^). The SEM images shown in Figure 3c before and after cycling also demonstrate a uniform Zn stripping/plating process for the nano-CaCO_3_-coated anode. Similarly, TiO_2_ [39] and Kaolin (Al_2_(Si_2_O_5_)(OH)_4_) coatings [40] were also constructed on the surface of the Zn anode. As shown in Figure 4a, in contrast to the electrodeposition behavior of bare Zn, under the elastic confinement of the TiO_2_/PVDF layer, Zn prefers to electrodeposit beneath the TiO_2_/PVDF layer and grow uniformly. Consequently, no protrusions or dendrites are observed on the surface of the TiO_2_/PVDF-Zn plate, even after 30 min of plating. As for the Kaolin coating, owing to the selective migration of Zn^2+^ in the layered channels of Kaolin and the assistance of adsorption sites such as Si-O and O-H bonds, the Kaolin coatings successfully guided the uniform plating of the Zn on Zn anode. When coupled with a MnO_2_ cathode, the Kaolin-Zn electrode presented a well-maintained morphology after 600 cycles at 0.5 A g^−1^, while many accumulations and protrusions existed on the surface of the bare Zn electrode.

Apart from the fabrication of ceramic coatings by using commercialized particles and a polymer binder, some more exquisite coatings were also constructed on Zn anodes for better electrochemical performances. Zhou and co-workers reported the fabrication of three-dimensional (3D) nanoporous ZnO coatings on the surface of Zn metal by an in situ deposition processes of Zn(OH)_2−_ onto Zn (Figure 4b) [41]. As shown in Figure 4b, according to first-principle calculations, the electrostatic attraction of 3D ZnO architecture towards Zn^2+^ can accelerate the kinetics of Zn^2+^ transfer and deposition by mitigating both the deposition barrier and the Zn^2^^+^ de-solvation energy barrier, thereby reducing energy consumption. The fabricated Zn@ZnO-3D/MnO_2_ battery can exhibit a specific capacity of 213 mAh g^−1^ after 500 cycles at 0.5 A g^−1^ with no capacity fading. In another study, an Al_2_O_3_ layer was engineered on the surface of a Zn plate by atomic layer deposition (ALD) [42]. As a result, a better surface wettability between Zn and the electrolyte was achieved, which could lead to a more even Zn^2+^ flux near the surface of the Zn plate. The formation of Zn dendrites was thus suppressed, and the corrosion was restrained. In a Zn|Zn symmetrical cell built with an Al_2_O_3_@Zn electrode, the electrode was characterized by a low overpotential of 36.5 mV. Apart from metal oxide coatings, Choi et al. engineered a novel ultrathin MoS_2_ interfacial layer to modify the Zn anode surface through an electrochemical deposition method for better electrochemical performances (Figure 4c) [43]. The vertically aligned MoS_2_ nanosheets guide Zn^2+^ flux and homogenize the surface electric field, which promotes homogeneous Zn plating/stripping and effectively suppresses dendrite formation. Furthermore, the MoS_2_ coating also improved the anodic diffusion processes, which was evidenced by EIS analysis. The rechargeable batteries consisting of a MoS_2_-Zn anode and MnO_2_ cathode displayed a high specific capacity of 638 mAh g^−1^ at 0.1 A g^−1^, and high stability of the Zn anode without Zn dendrites after 2000 cycles.

The solid electrolyte interphase (SEI), a film spontaneously formed on electrode surfaces in lithium-ion batteries, acts as a protective layer that enhances electrode stability during repeated cycling. Enlightened by this approach, fabricating artificial SEI protective layers on the surface of the zinc anode may also be beneficial for the electrochemical performances of the zinc anode. By manipulating the ion redistribution and providing physical isolation, the built SEI film could act as physical/chemical barrier to relieve the side reactions and dendrite growth during repeated cycling processes, enhancing the stability of zinc metals for long durability in Zn-Mn batteries [44].

In a recent study, Hu and colleagues designed and fabricated an indium-based artificial interphase on the Zn anode, which was shown to minimize interfacial resistance with the aqueous electrolyte and thereby inhibit Zn corrosion throughout cycling [45]. The In-based layer was achieved by the simple ion exchange reaction between In^3+^ and Zn to obtain In and Zn^2+^. Further XPS analysis indicated the existence of In_2_O_3_ and In(OH)_3_ on the superficial layer of In film, which was derived from the oxidation of In in air. It should be noted that the In_2_O_3_ and In(OH)_3_ containing coating film can act as a buffer layer and provide a potential gradient to drive the diffusion of Zn^2+^ through the coating layer, thus enabling the deposition of Zn underneath the layer. As a result, the symmetric cells with In-based layer-coated Zn anodes maintained excellent stability during 1400 h plating/stripping processes. Another artificial SEI film consisting of MOF and PVDF was fabricated on a Zn anode by Liu et al. through a slurry-coating process [46]. The hydrophilic microporous-rich UiO-66 MOFs enable a nanowetting effect with the Zn anode to ensure a sufficient contact between solid and liquid, thus leading to a reduced interfacial charge-transfer resistance and also a uniform Zn deposition process. Apart from pristine MOFs, MOF-derived porous carbon coatings can also serve as protective layers for zinc anode [47]. During this fabrication, the surface of the Zn anode was selectively oxidized to firstly form a ZnO layer, which was then coordinated with 2-MIM to form a ZIF-8 layer by a wet chemistry method. After a quick-calcination process, the ZIF-8 layer was then transformed into a hydrophilic N-doped porous carbon layer, which facilitated Zn^2+^ ion diffusion and uniform charge distribution, thereby effectively suppressing zinc dendrite formation and consequently enabling a highly reversible Zn plating/stripping process.

Table 1 summarizes the electrochemical performances of Zn-Manganese oxide batteries with functional coatings on the Zn anode.

### 3.2. Structure Optimization of Zn Anode

The microstructure and geometry of the Zn anode play a critical role in determining its electrochemical performance and stability during repeated plating/stripping cycles. For instance, compared with the Zn plate with a smooth surface, a well-engineered 3D zinc anode architecture, by providing a significantly larger surface area, distributes the current density at deposition sites, thereby effectively mitigating zinc dendrite formation [48]. In addition, some introduced secondary metal atoms around Zn atoms can also help regulate the Zn plating or enhance the anti-corrosion capability of Zn metals, finally resulting in greatly enhanced Zn plating/stripping electrochemical behavior [49].

**Figure 5:**
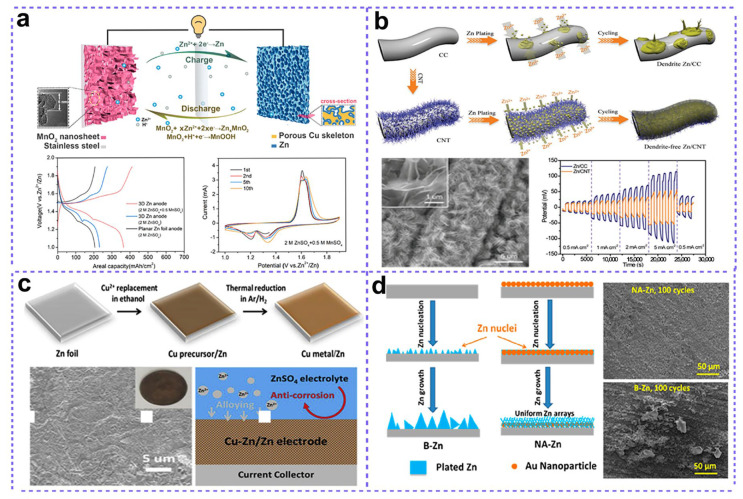
(**a**) Schematic and electrochemical performance of 3D Zn anode/MnO_2_ nanosheet cathode full cell. Adapted from [50], with permission from *ACS Sustainable Chem. Eng.*, 2019. (**b**) Fabrication of MnO_2_ nanosheets on the CNTs/CC substrates. Adapted from [53], with permission from *Adv. Mater.*, 2019. (**c**) Chemically resistant Cu–Zn alloy for Zn anode. Adapted from [54], with permission from *Energy Storage Materials*, 2020. (**d**) Quasi-isolated Au particles to guide uniform Zn deposition. Adapted from [55], with permission from *ACS Appl. Energy Mater.*, 2019.

Fabricating 3D zinc anodes by electrodepositing Zn on 3D conductive substrates has been drawing more and more attention recently. In 2019, Xu and co-workers fabricated a 3D zinc anode by electrodepositing zinc onto a chemically etched, porous 3D copper scaffold (Figure 5a) [50]. A planar copper foil was firstly immersed inside NH_3_·H_2_O solution to obtained a 3D porous copper matrix, which was then employed as a 3D conductive substrate for the electrodeposition of 3D Zn through a galvanostatic electrodeposition method. Owing to the open pore structure and excellent conductivity of the 3D porous copper substrate, the as-obtained 3D Cu/Zn anode achieved the uniform plating/stripping of Zn, thus resulting in a stable cycling performance, reduced polarization, and high coulombic efficiency up to almost 100% for 350 h. Similarly, some other metal substrates, such as Cu foam, Ni foam, and Cu foil, were also employed as substrates for the electrodeposition of Zn to fabricate Zn anodes [51]. In addition, carbon-based 3D substrates, which were widely used in various energy-related applications due to their light weight, low cost, corrosion resistance, and good mechanical properties, also showed great potential for fabricating 3D Zn anodes [52]. For example, Zeng and co-workers constructed a 3D Zn anode based on carbon cloth (CC) [53]. Specifically, a carbon nanotube (CNT) interconnected network was firstly fabricated on the surface of CC through a chemical vapor deposition method, which was then employed as a substrate for the subsequent electrodeposition of Zn nanosheets around the CNTs (Figure 5b). The CNT skeleton, with good conductivity and a high specific surface area, endows the constructed Zn/CNT anode with limited local current density, homogenous electric field distribution, and the low nucleation overpotential of Zn. These advantages synergistically mitigated dendrite formation and suppressed deleterious side reactions, leading to a stable cycling performance of 200 h with a 28.8% depth of discharge.

Apart from the geometry optimization of Zn anodes, composition regulation may also help boost the stability of Zn anodes. Composition regulation generally means the introduction of secondary metals in the Zn anode, which can improve the chemical resistance of the Zn anode during cycling processes, as well as modulate the Zn deposition behavior to suppress Zn dendrites during repeated plating/stripping. Recently, Cai et al. reported the introduction of chemically inert Cu to enhance the corrosion resistance of Zn anodes (Figure 5c) [54]. During this fabrication, the Cu/Zn composite was achieved by an ion exchange reaction between Zn and Cu^2+^ in ethanol, followed by a calcination process. The initially homogeneous and dense Cu/Zn composite was electrochemically reconfigured into a multiphase architecture comprising a Cu/Zn alloy interspersed with Zn metal during repeated charge–discharge cycles, which exhibited an extremely stable electrochemical plating/stripping process for more than 1500 cycles. In another similar work, the sputtering-deposited homogenous nano-Au particles on the Zn anode acted as heterogeneous seeds to induce the uniform plating/stripping processes of Zn on the anode (Figure 5d), guiding the formation of Zn-flake-array thus forbidding the generation of Zn dendrites. This simple Au-sputtering treatment of Zn anodes resulted in a prolonged cycling life for both Zn|CNTs/MnO_2_ batteries (from 48 to 2000 cycles) and Zn|Zn symmetric cells (from 92 to 1000 h) [55].

Table 2 summarizes the electrochemical performances of Zn-Manganese oxide batteries with structure optimization of Zn anodes.

## 4. Mn-Based Cathodes Design

For the cathode materials of rechargeable Zn-Mn batteries, various Mn-based oxides have been intensively investigated in recent years. As a typical transitional metal element, Mn can compound with different numbers of oxygens to form a series of multivalent phases, such as MnO, Mn_2_O_3_, MnO_2_, and Mn_2_O_7_. These manganese-based compounds often exhibit different electrochemical behaviors when employed as cathode materials for Zn-Mn batteries, owing to their various crystal structures and morphologies. Generally, several issues of Mn-based cathodes need to be addressed for the further development of Zn-Mn batteries. (i) The phase transformation during repeated electrochemical processes, such as the repeated insertion/extraction of Zn^2+^ in some cathodes during charging/discharging, can lead to large volume change and structural collapse, finally resulting in continuous capacity decay during cycling. (ii) The dissolution of Mn into the electrolyte during cycling can lead to the loss of active materials and then the capacity fading during cycling. (iii) The semi-conductive nature of manganese oxides hinders the electron transfer process during electrochemical performances, leading to a bad rate capability. In order to solve these problems, many effective strategies were developed which greatly promote the development of manganese-based cathodes. According to structural engineering strategies, the recent developments of Mn-based compounds are divided into four groups in this section, which include (i) metal doping in the Mn-based compounds, (ii) defect introducing, (iii) geometry and morphology engineering, and (iv) integration with functional materials. Both the synthetic strategies and the promotion mechanisms towards electrochemical activities are summarized in this section.

### 4.1. Metal Doping in Mn-Based Compounds

Doping modification, as an effective strategy for tailoring the electrochemical performance of MnO_2_, enables multiple effects through the introduction of guest ions: on the one hand, it can effectively suppress structural collapse and pulverization caused by phase transformations during cycling, thereby significantly enhancing structural stability; on the other hand, by modulating the electrostatic repulsion between Zn^2^^+^ and the host material, it optimizes ion insertion/extraction kinetics, thereby improving cycling stability and electrochemical reversibility. In doping modification studies, guest ions primarily stabilize the crystal structure through synergistic coordination bonding interactions with host atoms, with typical dopant ions including Na^+^, K^+^, V^5^^+^, Co^2^^+^, Cu^2^^+^, Zn^2^^+^, Ag^+^, and Ce^4^^+^.

**Figure 6:**
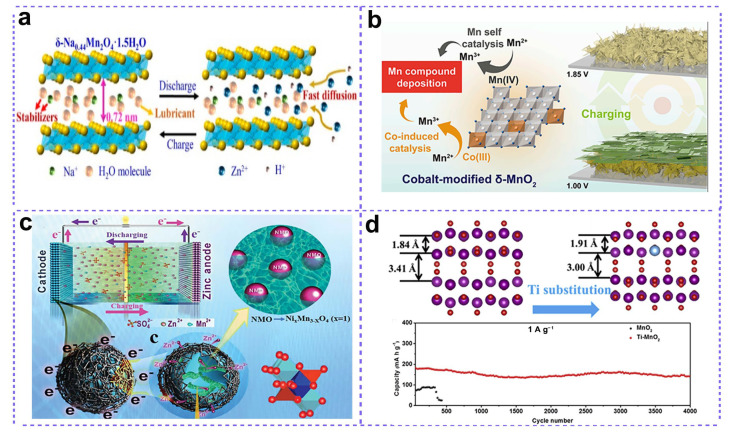
Metal ions doping for high-performance MnO_2_ cathode. (**a**) Na ions. Adapted from [56], with permission from *ACS Nano*, 2019. (**b**) Co ions. Adapted from [65], with permission from *iScience*, 2020. (**c**) Ni ions. Adapted from [66], with permission from *J. Mater. Chem. A*, 2019. (**d**) Ti ions. Adapted from [67], with permission from *Nano Energy*, 2019.

As discussed above, the slow Zn diffusion efficiency and the structural degradation of Mn-based oxides during repeated discharging/charging processes can result in kinetic limitations and inadequate cycle life, greatly hindering the development of ZMBs. For example, Zhi and co-workers proposed a pre-intercalation method to achieve a stable structure of δ-MnO_2_ by introducing Na ions and water molecules inside the MnO_2_ matrix (Figure 6a) [56]. The pre-intercalated δ-MnO_2_ was obtained by the oxidation reaction of Mn(OH)_2_ nanoplates inside a NaClO solution, with Na^+^ and water molecules intercalated inside the δ-MnO_2_ structure. When employing a Zn plate as an anode and 2M ZnSO_4_ and 0.2 M MnSO_4_ as electrolytes, the assembled ZMB coin cell can deliver a high reversible capacity of 278 mAh g^−1^ at 1 C, accompanied with an excellent rate capability of 106 mAh g^−1^ at 20 C and superlong cycling life up to 10,000 cycles. For comparison, the δ-NMOH sample with the thermal removal of water exhibited a far inferior electrochemical performance, indicating the important role of water molecules in structural stability and Zn^2+^ ion transportation. In addition to Na ions, some other alkaline ions were also proven to possess a similar function, including K^+^ and Ca^2+^ [57,58,59,60]. These pre-intercalated alkaline ions and other small molecules (such as water) not only act as pillars to enhance the structural integrity of MnO_2_, but also expand the Zn^2+^ immigration channels for better reaction kinetics, indicating that the alkaline-ion containing manganese oxides are potential cathode materials for ZMBs [61].

Apart from alkaline metal ions, transitional metal ions were also proven to be beneficial for improving the electrochemical performances in some respects, including the catalyzing effect towards the electrochemical deposition of Mn compounds, the enhancement of electrical conductivity and structural stability, the inducing of oxygen vacancy to facilitate ion transport, and so on [62,63,64]. These effective methods endow the Mn-compound cathodes with good stability, high specific capacity, outstanding rate capability, and long durability for ZMBs. For example, Shao’s group recently reported the dynamic self-recovery chemistry of manganese compounds as cathodes in ZMBs [65]. The fabricated Co-modified δ-MnO_2_ with Co-containing species highly dispersed into defective δ-MnO_2_ nanosheets displayed a high specific capacity of 500 mAh g^−1^, accompanied with an excellent capacity retention of 63% after 5000 cycles. Further investigations revealed that Co-species that bound on Mn-compounds can catalyze the deposition of Mn oxides during charging. Interestingly, the Co-species exhibited self-recovery behavior during electrochemical processes, which showed a reversible dissolution/deposition process (Figure 6b) during charge/discharge processes induced by the change in PH value due to the extraction/insertion of H^+^ in MnO_2_. In another study, by the substitution of Mn ions with Ni ions, Guo et al. fabricated a series of Ni-containing Mn-oxides, Ni_x_Mn_3_-_x_O_4_ (Figure 6c) [66], and the obtained NiMn_2_O_4_@C composite with an optimized Ni concentration exhibited outstanding Zn storage performance (129 mAh g^−1^ at 0.4 A g^−1^ after 850 cycles) due to the decreased band gap and enhanced electronic conductivity of the crystal. Ti ions were also employed to dope α-MnO_2_ for long-cycle ZMBs [67]. Further investigations showed that the introduction of Ti into α-MnO_2_ resulted in the contraction of tunnels in MnO_2_ (Figure 6d); however, this induced oxygen vacancy simultaneously, thus forming a charge depletion zone and creating a built-in electric field, which can facilitate charge transfer processes for better electrochemical performances.

Table 3 summarizes the electrochemical performances of Zn-Manganese oxide batteries with metal doping in cathodes.

### 4.2. Defect Engineering in Mn-Based Cathodes

Defect engineering is an effective method for inducing new properties that facilitate electrochemical processes, which is widely used in various energy-related fields, such as metal-ion batteries, electrocatalysis, and solar cells. Defect engineering generally involves some strategies to break the ordered arrangement in the original crystals, which includes the introduction of oxygen defects, cationic defects, amorphous structures, and so on [68]. The introduction of defects often results in more active sites inside the active materials, as well as optimization of the electronic structure for high charge transfer efficiency and proper ion adsorption capability, leading to greatly enhanced electrochemical performances [69].

**Figure 7:**
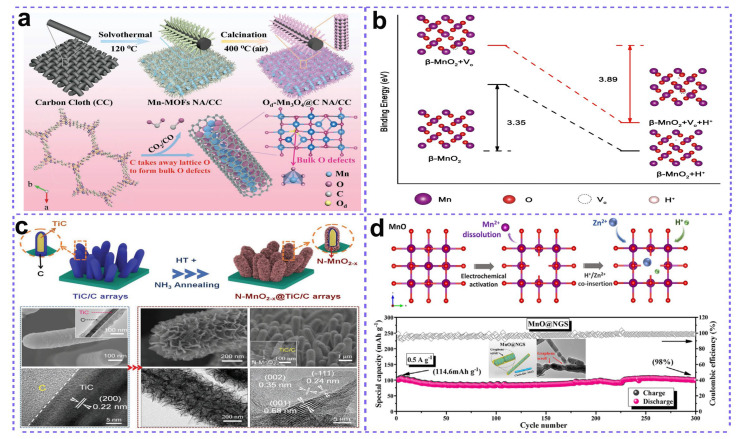
Defect engineering for high-performance manganese oxides cathodes. (**a**) Oxygen defects in Mn_3_O_4_. Adapted from [70], with permission from *Adv. Energy Mater.*, 2020. (**b**) Oxygen defects in MnO_2_. Adapted from [72], with permission from *iScience*, 2020. (**c**) N doping in MnO_2-x_. Adapted from [73], with permission from *Small*, 2019. (**d**) Cationic defects in MnO. Adapted from [78], with permission from *Chemical Engineering Journal*, 2020.

Oxygen defects, regarded as the creation of oxygen vacancies through calcination-driven lattice oxygen loss, have recently drawn much attention for fabricating high-performance cathode materials for ZMBs. Tan et al. reported the construction of oxygen-defect-rich Mn_3_O_4_@C nanorod arrays on carbon cloth (O_d_-Mn_3_O_4_@C NA/CC), which was derived from a fabricated Mn-MOF@CC as precursor (Figure 7a) [70]. It should be noted that the carbon element inside the Mn-MOF precursor plays an important role during the formation of effective O_d_-Mn_3_O_4_@C NA/CC cathodes, which not only consume lattice oxygen to form oxygen defects during calcination, but also generate a carbon matrix to provide conductive networks for charge transfer. First-principle calculations reveal that the induced oxygen defects increase the intrinsic conductivity of Mn_3_O_4_, as well as provide more active sites for Zn^2+^ and H^+^ insertion/extraction. As a result, the O_d_-Mn_3_O_4_@C NA/CC electrode exhibited an ultra-long cycling lifespan, delivering 84 mAh g^−1^ after 12,000 cycles at a high current density of 5 A g^−1^. In another work [71], the team demonstrated that oxygen defects reduce the Gibbs free energy required for Zn^2+^ adsorption, leading to enhanced reversibility of Zn^2+^ insertion/extraction in the defective MnO_2_ structure. The reduced electron demand for Zn-O formation in defective MnO_2_ liberates more electrons into the delocalized cloud, enhancing the attainable capacity and ultimately yielding excellent electrochemical performance as a ZIB cathode material. Interestingly, Zhou’s group proved that the β-MnO_2_ with abundant oxygen defects (D-β-MnO_2_) showed a lower binding energy of H^+^ insertion into the MnO_2_ matrix than the normal β-MnO_2_ (Figure 7b), which exhibited a high capacity of 302 mA h g^−1^ with a capacity retention of 94% after 300 cycles [72].

In addition to the aforementioned oxygen defects, the concurrent inducing of secondary nonmetal elements (such as N, P) can further modulate the electronic structure of Mn-based compounds so as to greatly benefit the electrochemical behaviors. Zhang et al. constructed a novel nitrogen-doped MnO_2-x_ with oxygen defects through a NH_3_ treatment process under low temperature (200 °C) (Figure 7c) [73]. During this fabrication, MnO_2_ nanosheet branches were firstly deposited on the surface of a TiC/C nanorod array backbone. A synergistic effect of nitrogen doping and oxygen defect creation was induced by NH_3_ treatment, leading to elevated electron densities and a lowered band gap in MnO_2_. The obtained N-MnO_2-x_ with rich oxygen vacancies and N-doping displayed an enhanced electronic conductivity and electrochemical activity, exhibiting faster reaction kinetics, a higher reversible capacity of 285 mAh g^−1^ at 0.2 A g^−1^, and a better long-cycling stability than the normal MnO_2_ counterparts. In a similar work, the same group also found that PO_4_^3−^ ions can be intercalated inside the MnO_2_ through a facial phosphorization process by using NaH_2_PO_2_ at 200 °C, which simultaneously introduced a lot of oxygen vacancies [74]. The oxygen vacancies can increase the electronical conductivity of MnO_2_ to facilitate charge transfer, while the phosphate ions can enlarge the interlayer spacing to accelerate the ion transport. As a result, the obtained P-MnO_2-x_@VMG cathode exhibited a high capacity retention of 91.3% after 1000 cycles at 2 A g^−1^, much better than that of the MnO_2_@VMG counterpart.

Apart from the above-mentioned oxygen defects, the introduction of cationic defects was also proven to be an effective method for modifying electronic structures in order to induce new properties [75,76,77,78]. For instance, the cationic defects in anatase TiO_2_ can act as intercalation sites for reversible Mg^2+^ and Al^3+^ insertion, allowing a much higher reversible capacity than that of pure TiO_2_ [75]. Similarly, cationic defects can also be introduced into MnO for better electrochemical performance for ZMBs. Zhou’s group reported an in situ electrochemical approach to induce Mn defects inside MnO during the first charging process, during which some Mn ions could be electrochemically extracted from the MnO matrix to form Mn defects [77]. The induced Mn defects were demonstrated by the increased Mn ratio in the electrolyte, as well as the obviously weak or missing Mn columns in HRTEM images. Due to the introduction of a large number of Mn defects, the obtained Mn_0.61_□_0.39_O (□ stands for Mn defects) has more accessible channels for ion transport during electrochemical processes. More importantly, the Mn_0.61_□_0.39_O cathode exhibited a Zn^2+^ extraction/insertion mechanism without structural collapse during repeated electrochemical processes, indicating an excellent cycling stability and high reversible capacity of 116 mAh g^−1^ after 1500 cycles at 1 A g^−1^. Zhao and co-workers also noticed the activation process of MnO with the forming of Mn defects during the first charging processes (Figure 7d) [78]. Moreover, they also found that the concentration of MnSO_4_ in the electrolyte has a great effect on the initial capacity and the following cycling stability, which suggests that the activation of MnO is ascribed to the dissolution of Mn into the electrolyte during the first charging process. In another study, MnS was found to display a similar activation process [79], which was transferred into manganese oxide (MnS-EDO) with rich defects during the charging process. The obtained MnS-EDO, possessing a large number of electrochemically active sites and fast ion diffusion kinetics, displayed a high specific capacity of 336 mAh g^−1^ after 100 cycles with a high capacity retention of nearly 100% at 0.3 A g^−1^.

Amorphous materials, which present a short-range order with irregular intervals, recently have drawn much attention for their applications in energy storage fields. On the one hand, the abundant structural defects in the amorphous electrode materials can provide more active sites for ion storage, as well as improve ion diffusion through the lattice to enhance the reaction kinetics. On the other hand, the intrinsically isotropic nature of amorphous materials can help alleviate the strain generated from ion insertion and form a lower volume expansion, thus ensuring good cycling stability without significant structural collapse. Owing to these unique advantages, amorphous materials exhibit excellent electrochemical performances in various energy storage and conversion devices, including Li-ion batteries, K-ion batteries, etc.

Srinivasan and co-workers recently found amorphous MnO_2_ (A-MnO_2-δ_) to be a potential cathode material for ZMBs [80]. The abundant structural defects in A-MnO_2-δ_ nanospheres and their isotropic nature ensure good reaction kinetics and electrochemical activities induced by the short Zn^2+^ diffusion path and fast diffusion kinetics, endowing A-MnO_2-δ_ with a high reversible capacity of 301 mAh g^−1^ at 100 mA g^−1^ and high capacity retention of 78% after long cycling of 1000 cycles. Moreover, the reaction mechanism of this A-MnO_2-δ_ was explored by an in situ XRD technique. The A-MnO_2-δ_ shows four stages during one discharge–charge process. At the first stage between 1.85 V to 1.3 V, no noticeable peaks can be observed, indicating that the initial Zn^2+^ insertion inside the lattice does not change the amorphous nature of A-MnO_2-δ_. During the following second stage between 1.3 V to 1.0 V, several peaks assigned to Zn_4_SO_4_(OH)_6.5_H_2_O are observed, suggesting the insertion of H^+^. During the charging process, the peaks of Zn_4_SO_4_(OH)_6.5_H_2_O disappear in the third stage (1.0 V–1.5 V), and finally transfer into the initial amorphous condition again. The in situ XRD tests confirm the high reversibility of the amorphous A-MnO_2-δ_ cathode, as well as the reaction mechanism of Zn^2+^ and H^+^ co-insertion during electrochemical processes. Amorphous MnO_2_ nanosheets were further constructed directly on the 3D carbon nanotube foam through a simple deposition process, and the obtained free-standing 3D CNT/amorphous MnO_2_ can be directly employed as a flexible cathode for flexible ZMBs [81].

Table 4 summarizes the electrochemical performances of Zn-Manganese oxide batteries with defect engineering in cathodes.

### 4.3. Structural Optimization

For the electrochemical reactions on the electrodes, a whole electrochemical process generally involves the transport of reactants onto the active sites, then the reaction on active sites, and finally the transport of products into the surrounding electrolytes. The complicated electrochemical procedures indicate that the final electrochemical performances not only depend on the intrinsic activity of active sites, but also are influenced by some other parameters of the cathode materials, such as morphology, pore size, and dimension [82]. The structural optimization here means the optimization of these factors in order to maximize the electrochemical behaviors, which generally includes the engineering of hollow or porous structures, the fabrication of low-dimensional manganese oxides, the modulation of crystal structure, the optimization of wettability of cathode materials, and so on. All these optimization strategies can facilitate the electrochemical processes in some respects, such as by accelerating the transportation of reactants, providing large amounts of active sites, or shortening the ion transport path. The structural optimization strategies towards manganese-based cathodes are comprehensively summarized in this section.

**Figure 8:**
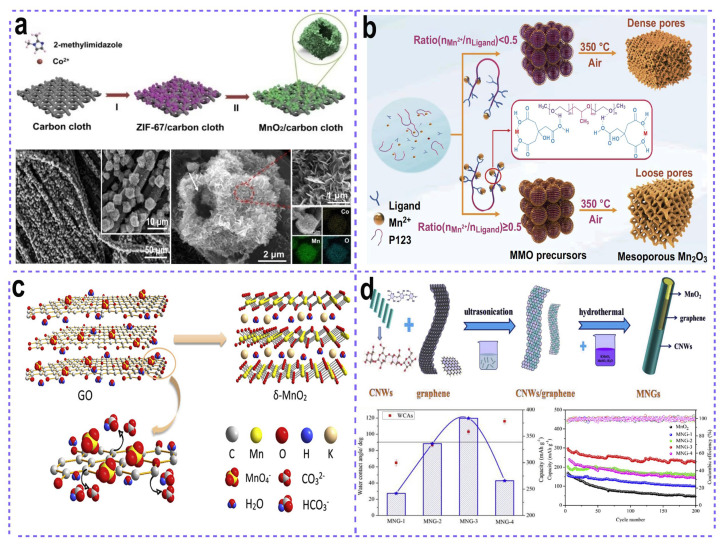
Structural optimization for effective manganese oxides cathodes. (**a**) MnO_2_ nanosheet-assembled hollow polyhedrons. Adapted from [83], with permission from *ChemSusChem*, 2020. (**b**) Inverse opal MnO_2_ with ordered macropores. Adapted from [84], with permission from *Nano Res*, 2019. (**c**) Graphene oxide-derived ultra-thin MnO_2_ nanosheets. Adapted from [88], with permission from *Electrochimica Acta*, 2019. (**d**) Adjusting the hydrophilic/hydrophobic of MnO_2_ cathode. Adapted from [90], with permission from *Energy Storage Materials*, 2020.

Porous materials, which possess large amounts of pores inside, have drawn great attention in the energy storage field. Compared with the normal solid bulk materials, porous materials have numerous advantages, such as larger specific surface area, tunable pore size, more exposed active sites, and accelerated mass transport. Thus, the rational fabrication of porous or hollow materials is highly important for improving electrochemical activities and kinetics so as to fabricate effective cathode materials for various energy devices, especially ZMBs. Cao proposed a facial synthesis of a hollow polyhedron assembled by MnO_2_ nanosheets through a mild-hydrothermal reaction by employing ZIF-67 as a sacrificing template [83]. During this process, due to the instability of ZIF-67 in solution at high temperatures, the ZIF-67 gradually dissolved, while at the same time a MnO_2_-nanosheet hollow polyhedron was gradually constructed based on the sacrificing of the ZIF-67 polyhedron (Figure 8a). Owing to the ultrathin nanosheet subunits that provide large amounts of exposed active sites for effective Zn^2+^ storage, as well as the open space between the nanosheets that facilitate the electrolyte penetration, the as-fabricated MnO_2_-nanosheet hollow polyhedral cathode exhibited a Zn^2+^ storage capability of 264 mAh g^−1^ after 300 cycles at 1 A g^−1^.

The fabrication of hollow or porous structures by employing sacrificing templates is commonly observed in the previous literature. The pore parameters, including pore size, pore shape, and pore volume, are highly controllable, which can be easily regulated by the employed sacrificing templates. Polystyrene (PS) has recently been widely applied in the fabrication of porous or hollow nanostructures [18]. PS spheres with controllable sizes are suitable for the construction of desired porosity inside nanostructures because their diameters are highly adjustable and they can be easily removed by calcination under high temperatures or dissolution in some organic polar solvents such as Dimethyl Formamide (DMF) or tetrahydrofuran (THF). Yan’s group fabricated an inverse opal manganese oxide consisting of ultrathin nanosheets based on a mild solution-based reaction by using PS as a template [84]. When the reduction reaction of KMnO_4_ was conducted in the weak acid solution, the layered structured birnessite MnO_2_ started to nucleate in the interspaces of PS spheres, resulting in the formation of an inverse opal MnO_2_ consisting of nanosheets. The electrode delivered an exceptional specific discharge capacity of 263 mAh·g^−1^ at a current density of 300 mA·g^−1^ over 100 cycles, with a high capacity retention of 95.6%—a performance originating from its unique architecture combining multi-layered nanosheets with an inverse opal framework. In another investigation, the pore structure of the Mn_2_O_3_ cathode is tunable via regulating the coordination degree between Mn^2^^+^ and citric acid ligands [85], with the pore size adjustable in the range of 3.2–7.3 nm and the BET specific surface area controllable from 55 to 260 m^2^·g^−1^ (Figure 8b). For the fabrication of porous MnO_2_, an alternative effective approach involves constructing MnO_2_ on porous substrates. Specifically, MnO_2_ nanotubes have been successfully synthesized through electrodeposition of a thin MnO_2_ layer onto porous nickel nanotube membranes [86]. Benefiting from the excellent mass and charge transfer conductivity, the Zn-MnO_2_ battery using MnO_2_/Ni nanotubes as the cathode exhibits a maximum power density of 1.38 kW·kg^−1^.

Two-dimensional (2D) materials, characterized by their single-layer thickness, have attracted considerable attention due to their extensively exposed surfaces and numerous active sites. Consequently, 2D MnO_2_ nanosheets are promising candidates for high-performance cathodes in aqueous zinc-ion batteries. By the ordered restacking of exfoliated MnO_2_ single atomic layers, Hu and co-workers constructed a two-dimensional δ-MnO_2_ nanofluidic channels for effective Zn ion transportation [87]. Dendrite inhibition is achieved by the abundant vertical and horizontal 2D nanochannels, which ensure a uniform distribution of Zn^2+^ ion flow by providing ample diffusion pathways. In a similar work, two-dimensional δ-MnO_2_ nanosheets with a thickness of about 2–4 nm were obtained by the in situ reduction of KMnO_4_ on two-dimensional graphene oxide (Figure 8c) [88]. The δ-MnO_2_ cathode delivered a reversible capacity of 133 mAh g^−1^ at 100 mA g^−1^ after 100 cycles, a performance attributed to its two-dimensional architecture, which provides a large surface area and a high density of accessible active sites.

According to the previous literature, MnO_2_ possesses several different phases, including vernadite, pyrolusite, nsutite, and so on. Different MnO_2_ phases with different crystal structures will influence the insertion/extraction processes of Zn^2+^, thus greatly affecting the electrochemical performances. Pan’s group observed the phase evolution from the initial mixture of vernadite, pyrolusite, and nsutite to a final pyrolusite phase when the microwave hydrothermal time exceeds 120 min [89]. Further investigation shows that the pyrolusite phase with high Mn valence, high structural stability, and low BET surface exhibited a better electrochemical performance compared with other MnO_2_ phases. By engineering the blend ratio of hydrophobic graphene to hydrophilic cellulose nanowhiskers, the cathode’s wettability was tailored to probe its influence on electrochemical behavior (Figure 8d) [90]. The MnO_2_ cathode with a proper wettability (exhibiting a contact angle of 103.04 ± 2.91°) can display a high reversible capacity of 384 mAh g^−1^ and an ultra-long lifespan of 5000 cycles at 20 C.

Despite the unique structural advantages of nanoscale MnO_x_ with different dimensions (e.g., high specific surface area, short ion diffusion pathways, and abundant active sites), which have laid a crucial foundation for enhancing the structural stability and electrochemical performance of zinc-MnO_x_ batteries, their inherent challenges in electrochemical energy storage applications remain pressing issues to be addressed. On the one hand, during charge–discharge cycling, nanoscale MnO_x_ particles tend to agglomerate due to their high surface energy, thereby increasing internal ion/electron transport resistance within the electrode, reducing the utilization efficiency of active sites, and ultimately leading to rapid capacity fading. On the other hand, the excessive aggregation of nanoparticles easily forms dense packing structures, which not only restricts electrolyte wettability but also results in low volumetric energy density of the electrode material, failing to meet practical application requirements. Therefore, the rational design of nanoscale MnO_x_ must not only retain its nanoscale advantages to overcome the kinetic limitations of microscale materials but also involve integration with other materials or further optimization of its own structure to achieve a superior performance.

Table 5 summarizes the electrochemical performances of Zn-Manganese oxide batteries with structural optimization in cathodes.

### 4.4. Compositing with Conductive Agents

During the repeated charge–discharge cycles, diverse ions and electrons migrate between the cathode and anode, rendering charge transport a process that exerts a pivotal influence on electrochemical reactions. As a representative semiconductor material, MnO_2_—characterized by inherently low electrical conductivity—significantly impedes charge transport during electrochemical processes. In this context, constructing composites of MnO_2_ with conductive agents emerges as a promising strategy to facilitate charge transfer, thereby enhancing electrochemical performance.

Due to the excellent electrical conductivity of carbon-based materials, the composite of manganese oxide with carbon materials has emerged as a crucial approach to enhance its electrochemical performance [91]. In recent decades, researchers have attempted to combine manganese oxide with various carbon materials to accelerate the charge transport process during electrochemical reactions, yielding unexpected results. In this chapter, we will summarize the composite strategies of manganese oxide with different types of carbon materials and conduct a detailed analysis of the roles played by carbon materials.

**Figure 9:**
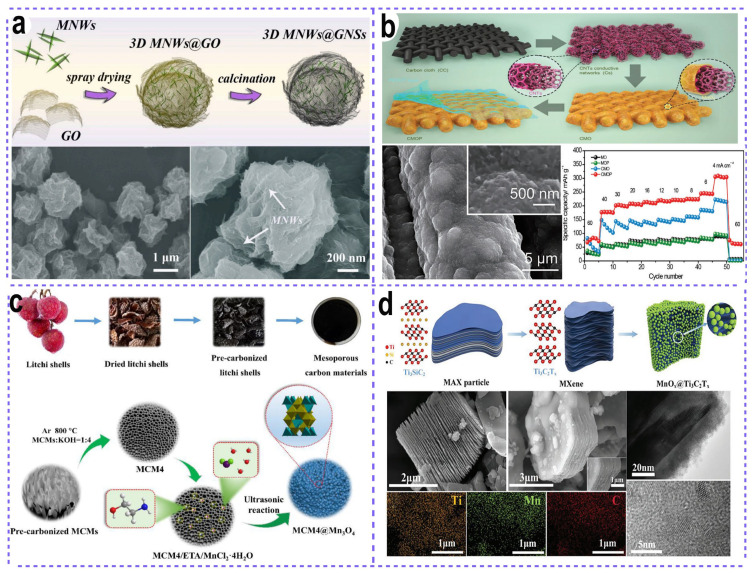
Constructing composites with conductive functional materials for high-performance manganese oxides cathodes. (**a**) 3D graphene nanosheet microflowers confining MnO_2_ nanowires. Adapted from [92], with permission from *Journal of Power Sources*, 2020. (**b**) 3D carbon nanotubes/carbon cloth networks for supporting MnO_2_. Adapted from [95], with permission from *Small Methods*, 2019. (**c**) Litchi shell-derived porous carbon for accommodating Mn_3_O_4_ particles. Adapted from [101], with permission from *Electrochimica Acta*, 2020. (**d**) 2D MXene nanosheets for loading MnO_x_ particles. Adapted from [109], with permission from *Adv. Funct. Mater.*, 2019.

The first type of material to be reviewed is three-dimensional carbon matrices, which can provide a large number of binding sites for the integration of manganese oxide across three-dimensional spatial dimensions. In some cases, the selection of three-dimensional self-supported substrates can even directly serve as binder-free self-supported cathodes. Three-dimensional carbon substrates generally include three-dimensional graphene nanosheets, three-dimensional carbon gels, carbon paper, carbon cloth, and so forth. Yan et al. reported the synthesis of a three-dimensional (3D) MnO_2_@graphene composite via a facile spray-drying method (Figure 9a), using a suspension of MnO_2_ nanowires and graphene oxide (GO) nanosheets as precursors [92]. The resulting 3D microflower-like architecture, with MnO_2_ nanowires confined within conductive graphene nanosheets, exhibited an exceptional rate performance and cycling stability (97.5% capacity retention after 1000 cycles). This superior behavior can be attributed to the structural integrity of its robust framework and the abundant void space within the composite, which effectively mitigates volume variation during electrochemical processes. Analogous structures can also be constructed through alternative approaches such as hydrothermal synthesis or simple filtration, yielding binder-free α-MnO_2_/graphene aerogel or MnO_2_/reduced graphene oxide (rGO) cathodes, respectively [93,94].

Commercial carbon substrates can also be utilized to load MnO_2_ for the fabrication of binder-free cathodes. These substrates typically include carbon cloth, carbon paper, and carbon fiber cloth. By providing three-dimensional (3D) highly conductive pathways for charge transportation, such carbon substrates effectively ensure excellent electrochemical performance. For instance, Lu et al. reported the design of carbon nanotube-decorated carbon cloth as a support for MnO_2_ deposition (Figure 9b) [95]. With the additional protection of a PEDOT layer, the resulting binder-free MnO_2_-based cathode exhibited a superior rate capability of 306 mAh g^−1^ under a high current density of 1.1 A g^−1^, accompanied by a high capacity retention of 81.3% after 2000 cycles. In another study, Gao and co-workers fabricated MnO_2_ nanosheet-assembled hollow polyhedral structures on carbon cloth for flexible zinc-ion batteries (ZIBs), using ZIF-67 polyhedrons as templates [83]. Owing to the robust structural integrity of the hollow architecture and the high conductivity of the carbon cloth substrate, the flexible Zn-MnO_2_ battery demonstrated a stable power supply to a light-emitting diode (LED), even under repeated bending and folding conditions. Beyond this, other carbon-based substrates have been explored in related works, including electrochemically derived graphene-like carbon films [96], carbon paper [97], and carbon fibers [98,99]. Notably, when carbon fibers were employed as supports for MnO_2_ cathodes, a cable-type Zn-MnO_2_ microbattery was successfully constructed.

Carbon coating has emerged as a widely adopted strategy for enhancing the electrochemical performance of active materials. The carbon layers encapsulating the active materials can function as protective barriers to mitigate agglomeration and structural collapse of the active components, while simultaneously facilitating charge transfer kinetics to ensure excellent rate capability. For instance, Ma et al. reported the synthesis of Mn_3_O_4_/carbon nanowire cathodes, where Mn_3_O_4_ nanoparticles were confined within carbon nanoreactors [100]. Benefiting from the carbon matrix, the Mn_3_O_4_@C nanowire cathode delivered a high reversible capacity of 380 mAh g^−1^. Zhou et al. developed a mesoporous carbon matrix embedded with Mn_3_O_4_ nanoparticles through a straightforward biomass conversion process, using litchi shells as the precursor followed by a Mn_3_O_4_ precipitation step (Figure 9c) [101]. Analogously, various other carbon-based protective coatings have been documented, including graphene scroll coatings [102], MOF-derived N-doped carbon coatings [103], and carbon nanotube coatings [104].

Beyond conductive carbon materials, other conductive substrates such as metal substrates [105] (e.g., stainless steel mesh), conductive polymers [106,107,108] (e.g., polypyrrole, polyaniline), and two-dimensional MXene [109] have also been utilized. For instance, MnO_x_ decorations were in situ formed on the surface of MXene nanosheets through the reaction between KMnO_4_ and fresh-obtained Ti_3_C_2_T_x_ nanosheets with abundant functional groups (Figure 9d). The highly conductive Ti_3_C_2_Tx nanosheets offer rapid transport channels for electron and ion transport, thereby ensuring an excellent rate performance, with 50% capacity retention upon a 100-fold increase in current density (from 0.1 to 10 A g^−1^). These substrates play analogous roles to carbon materials, not only enhancing charge transfer processes and protecting active materials but also providing buffering space to accommodate volume expansion. Owing to these significant advantages of supporting materials, MnO_2_-based electrodes have demonstrated remarkably improved electrochemical performance in Zn-ion batteries.

Table 6 summarizes the electrochemical performances of Zn-Manganese oxide battery cathodes composited with conductive agents.

## 5. Conclusions

In summary, this paper reviews the latest research progress in zinc–manganese oxide batteries, focusing on three core aspects: energy storage mechanisms, anode modification, and cathode enhancement strategies. First, it provides a systematic overview of the fundamental reaction mechanisms in Zn–MnO_2_ batteries, with a focus on Zn^2+^ intercalation, H^+^/Zn^2+^ co-intercalation, and the dissolution–deposition processes of manganese species. Regarding the dendrite growth and side reactions issues faced by the metallic zinc anode, this paper combs the regulatory strategies, such as anode protective layer construction, three-dimensional current collector design, and inducer surface modification. For the key challenges of the manganese oxide cathode, such as Mn dissolution and loss, slow ion intercalation/deintercalation kinetics, and poor electronic conductivity, this paper summarizes improvement methods, including heterogeneous metal ion doping, defect engineering, structural optimization, and combination with conductive substrates.

Despite significant progress in zinc–manganese oxide battery research, the precise elucidation of their electrochemical reaction mechanisms remains a core scientific bottleneck limiting performance enhancement. As reviewed in this paper, the energy storage mechanism of manganese-based cathode materials remains highly debated, with no consensus reached to date. Non-standardized experimental protocols and limitations of characterization techniques may lead to ambiguous conclusions, thereby hindering the rational design of high-performance cathode and anode materials. To address this challenge, there is an urgent need for the integration of multi-dimensional electrochemical methods, advanced characterization technologies, and precise theoretical calculations. For instance, emerging techniques such as in situ Raman spectroscopy and in situ X-ray absorption spectroscopy (XAS), combined with density functional theory (DFT) calculations and machine learning-assisted analysis, enable dynamic tracking of reaction intermediates and active sites [110]. The application of these advanced characterization tools will provide critical support for the accurate elucidation of zinc–manganese oxide battery reaction mechanisms and offer theoretical guidance for materials design.

The electrolyte system of zinc–manganese oxide batteries is a key underpinning of their safety advantages but faces the challenge of limited operating voltage. Typically employing aqueous electrolytes, this system significantly enhances battery safety by effectively mitigating fire and explosion risks associated with organic electrolytes in conventional lithium-ion batteries. However, the narrow electrochemical stability window of aqueous electrolytes (usually <2 V) results in low operating voltage, restricting the improvement of energy density [111]. As a core factor influencing battery performance, the research depth of electrolyte systems lags far behind that of electrode materials, and scientific issues such as interface regulation and ion transport therein urgently require in-depth exploration. Thus, the design and development of functional electrolytes (e.g., high-concentration electrolytes, gel electrolytes, and additive engineering) will emerge as crucial directions to break voltage limitations and optimize zinc-ion transport kinetics, warranting focused attention in future studies [25].

To meet the development demands of flexible electronics and wearable devices, the development of flexible/wearable and multifunctional zinc–manganese oxide batteries represents an important future research direction. Such batteries need to integrate flexible/wearable characteristics with multifunctional responses (e.g., self-healing, self-protection, and stretchability), providing a new paradigm for energy supply in next-generation smart electronic devices. The core challenge lies in constructing solid-state/quasi-solid-state polymer electrolytes with high ionic conductivity, excellent mechanical strength, and multifunctional properties (e.g., stretchability, compressibility, toughness, and self-healing capability). Additionally, interface compatibility between multifunctional electrolytes, smart materials, and other battery components must be enhanced through device structure optimization and intelligent design strategies to achieve efficient integration of multifunctional devices without significantly compromising electrochemical performance. Notably, the introduction of novel smart materials (e.g., stimuli-responsive polymers, conductive metal–organic frameworks) and the adoption of biomimetic design principles inspired by complex natural systems (e.g., the hierarchical structure of spider silk and dynamic response mechanisms of muscle tissue) will further expand the prototype development of flexible/wearable and multifunctional zinc–manganese oxide batteries, facilitating their transition from laboratory research to practical applications.

It is expected that this review will provide theoretical guidance for the future development of zinc–manganese oxide batteries and promote the industrialization of this system.

## Figures and Tables

**Table 1:** Electrochemical performance summary of Zn-Manganese oxide batteries with functional coatings on Zn anode.

Coatings on Zn Anode	Current Density	Specific Capacity (mAh g^−1^)	Cycling Numbers	Ref.
Nanoporous CaCO_3_	1 A g^−1^	177	1000 cycles	[38]
TiO_2_/PVDF	2 C	234	300 cycles	[39]
Kaolin (Al_2_(Si_2_O_5_)(OH)_4_)	0.5 A g^−1^	190	600 cycles	[40]
3D nanoporous ZnO	0.5 A g^−1^	212.9	500 cycles	[41]
Al_2_O_3_ layer	1 A g^−1^	158.4	1000 cycles	[42]
MoS_2_	0.1 A g^−1^	638	2000 cycles	[43]
In_2_O_3_/In(OH)_3_	1.5 A g^−1^	190	400 cycles	[45]
ZIF-8 derived carbon	0.1 A g^−1^	266.5	100 cycles	[47]

**Table 2:** Electrochemical performance summary of Zn-Manganese oxide batteries with structure optimization of Zn anode.

Materials	Current Density	Specific Capacity (mAh g^−1^)	Cycling Numbers	Ref.
3D Zn anode on 3D copper	0.4 A g^−1^	173	300 cycles	[50]
Zn on Cu foam	1 A g^−1^	207	500 cycles	[51]
Zn on CNTs/ carbon cloth	20 mA cm^−2^	167	1000 cycles	[53]
Cu/Zn composite	1 mA cm^−2^	46 mV overpotential	1500 cycles	[54]
Nano Au on Zn anode	0.5 A g^−1^	67	2000 cycles	[55]

**Table 3:** Electrochemical performance summary of Zn-Manganese oxide batteries with metal doping in cathodes.

Materials	Current Density	Specific Capacity (mAh g^−1^)	Cycling Numbers	Ref.
Na^+^ doped MnO_2_	20 C	106	10,000 cycles	[56]
K^+^ doped MnO_2_	5 C	180	400 cycles	[57]
Ca^2+^ doped MnO_2_	175 mA g^−1^	298	5000 cycles	[60]
Co^2+^ doped MnO_2_	300 mA g^−1^	435	100 cycles	[65]
NiMn_2_O_4_@C	0.4 A g^−1^	129	850 cycles	[66]
Ti^4+^ doped MnO_2_	100 mA g^−1^	225	200 cycles	[67]

**Table 4:** Electrochemical performance summary of Zn-Manganese oxide batteries with defect engineering in cathodes.

Materials	Current Density	Specific Capacity (mAh g^−1^)	Cycling Numbers	Ref.
O_d_-Mn_3_O_4_@C	5 A g^−1^	84	12,000 cycles	[70]
Od-MnO_2_	5 A g^−1^	105	100 cycles	[71]
Dd-β-MnO_2_	100 mA g^−1^	276	50 cycles	[72]
N doped MnO_2-x_	1 A g^−1^	173	1000 cycles	[73]
P doped MnO_2-x_	2 A g^−1^	186	1000 cycles	[74]
MnO with Mn defects	1 A g^−1^	116	1500 cycles	[77]
Amorphous MnO_2-δ_	1 A g^−1^	147	1000 cycles	[80]

**Table 5:** Electrochemical performance summary of Zn-Manganese oxide batteries with structural optimization in cathodes.

Materials	Current Density	Specific Capacity (mAh g^−1^)	Cycling Numbers	Ref.
Hollow polyhedron MnO_2_ nanosheets	1 A g^−1^	264	300 cycles	[83]
Inverse opal MnO_2_	300 mA g^−1^	263	100 cycles	[84]
Nanoporous Mn_2_O_3_	3.08 A g^−1^	146	3000 cycles	[85]
2D MnO_2_ nanosheets	100 mA g^−1^	274	600 cycles	[87]
2–4 nm δ-MnO_2_ nanosheets	100 mA g^−1^	133	100 cycles	[88]
Pyrolusite phase MnO_2_	4 C	134	1000 cycles	[89]
MnO_2_ with 103° contact angle	20 C	108	5000 cycles	[90]

**Table 6:** Electrochemical performance summary of Zn-Manganese oxide battery cathodes composited with conductive agents.

Materials	Current Density	Specific Capacity (mAh g^−1^)	Cycling Numbers	Ref.
3D MnO_2_@graphene	2 A g^−1^	192	10,000 cycles	[92]
MnO_2_/rGO	6 A g^−1^	172	500 cycles	[94]
MnO_2_/CNTs/CC	10.8 A g^−1^	177	2000 cycles	[95]
MnO_2_/carbon film	1 A g^−1^	188	1000 cycles	[96]
MnO_2_/carbon paper	1 A g^−1^	163	200 cycles	[97]
Mn_3_O_4_/carbon nanowire	10 A g^−1^	135	2000 cycles	[100]
Mn_3_O_4_/litchi shell-derived carbon	600 mA g^−1^	275	1000 cycles	[101]
MnO_2_/graphene scroll	3 A g^−1^	145	3000 cycles	[102]
Mn_3_O_4_/stainless steel mesh	500 mA g^−1^	296	500 cycles	[105]
Mn_2_O_3_/polypyrrole	400 mA g^−1^	178	2000 cycles	[108]
MnO_2_/MXene	5 A g^−1^	130	400 cycles	[109]
